# A Rare Retrorectal Dilemma: Tailgut Cyst Uncovered and Excised

**DOI:** 10.7759/cureus.116070

**Published:** 2026-09-10

**Authors:** Ashtha Singh, Mohammad Saquib Ali, Sanjay Kala

**Affiliations:** 1 General Surgery, Ganesh Shankar Vidyarthi Memorial (GSVM) Medical College, Kanpur, IND

**Keywords:** mri diagnosis, presacral cyst, retro rectal hamartoma, surgical excision, tailgut cyst

## Abstract

Tailgut cysts are uncommon congenital lesions that develop from remnants of the embryonic hindgut. They are most frequently found in middle-aged women. This report describes a 35-year-old Indian woman who presented with a long-standing history of a painless, slowly enlarging swelling in the gluteal region. Physical examination identified a large, fluctuant mass without signs of inflammation. Pelvic magnetic resonance imaging (MRI), which is the preferred diagnostic method, revealed a multiloculated cystic lesion in the presacral space. Characteristic imaging findings included internal septations and no connection to adjacent structures. The cyst was completely removed via a retrococcygeal surgical approach. Histological examination confirmed the diagnosis of a benign tailgut cyst, showing a lining of various epithelial types with no evidence of cancer. The patient recovered well after surgery and remained asymptomatic, with no clinical evidence of recurrence, at 18 months of follow-up. This case highlights that tailgut cysts should be considered in the differential diagnosis of presacral and gluteal masses. MRI is essential for preoperative evaluation and to distinguish these cysts from other entities. Complete surgical removal is the standard treatment to prevent potential complications such as infection, recurrence, or malignant change. The retrococcygeal approach is highly successful, and timely excision is associated with a favorable outcome.

## Introduction

Tailgut cysts, also known as retrorectal cystic hamartomas, are rare developmental lesions arising from persistent remnants of the embryonic hindgut, with an estimated incidence of one in 40,000 hospital admissions [1]. These cysts are most frequently diagnosed in middle-aged women (30-50 years of age) and are characteristically located in the presacral space [2]. Embryologically, they originate from the incomplete involution of the tailgut, a transient structure distal to the hindgut that normally regresses by the 8th week of gestation [3]. This pluripotent origin is reflected in their histopathology, as the cysts are typically lined by a heterogeneous combination of epithelial tissues, including squamous, transitional, and glandular cells [4].

Clinically, a significant proportion (30-50%) of tailgut cysts remain entirely asymptomatic and are discovered incidentally [5]. When symptomatic, common presentations include pelvic or perianal discomfort (noted in approximately 42% of cases), gluteal swelling (35%), bowel dysfunction due to mass effect (23%), and complications such as infection or fistula formation (15%) [6,7]. Because these features overlap with far commoner perianal conditions such as pilonidal disease, perianal abscess, and lipoma, the correct diagnosis is often reached late, sometimes only after repeated treatment for a presumed simple perianal problem.

Magnetic resonance imaging (MRI) is the gold standard for diagnosis, reliably demonstrating a multiloculated cystic lesion in the presacral space. Key diagnostic features on MRI include high T2-weighted signal intensity (93%), internal septations (67%), and the definitive presacral location (100%) [8]. Complete surgical excision is the definitive treatment and is imperative to prevent local complications and to eliminate the risk of malignant transformation, which is reported in 3-5% of cases [9]. The diagnosis is ultimately confirmed by histopathological examination, which reveals the characteristic heterogeneous epithelial lining [4,10].

The purpose of this report is to describe the clinical, radiological, operative, and histopathological features of a large retrococcygeal tailgut cyst in a young woman in whom the lesion had been present for five years, to set out how it can be separated from the common causes of gluteal and presacral swelling, and to explain the reasoning behind the posterior retrococcygeal approach used for its complete excision.

## Case presentation

In October 2024, a 35-year-old Indian woman of South Asian ethnicity, resident in Kanpur, Uttar Pradesh, India, presented to the Department of General Surgery, Ganesh Shankar Vidyarthi Memorial (GSVM) Medical College and Lala Lajpat Rai Hospital, Kanpur, Uttar Pradesh, India, with a gradually enlarging swelling in the gluteal cleft region over five years, associated with mild discomfort but no history of trauma, prior surgeries, or systemic illness. Clinical examination revealed a well-defined, nontender, fluctuant swelling in the left gluteal region without signs of inflammation, discharge, or neurological deficits (Figure 1). Digital rectal examination was unremarkable.

**Figure 1:**
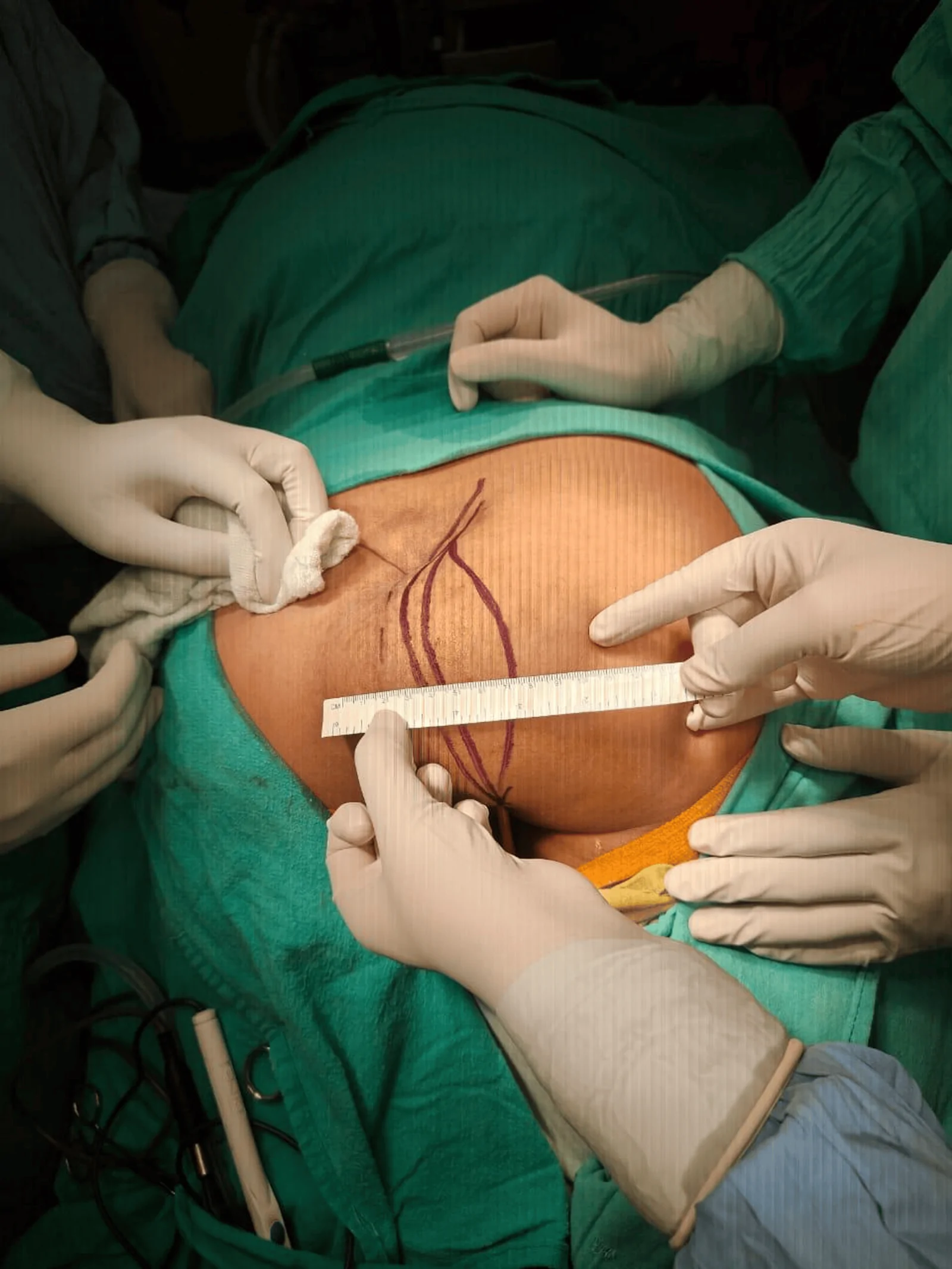
Left gluteal swelling Photograph taken in the operating theater before incision, showing a well-defined, dome-shaped, nontender, fluctuant swelling in the left gluteal region extending toward the gluteal cleft. The overlying skin is normal, with no erythema, sinus opening, discharge, or scarring. Surface markings outline the planned incision, and a scale is held against the swelling to demonstrate its size

MRI revealed a 10 × 9 cm T2-hyperintense cyst displacing the rectum, consistent with a tailgut cyst (Figure 2) [3]. Surgical excision via the retrococcygeal approach achieved complete resection.

**Figure 2:**
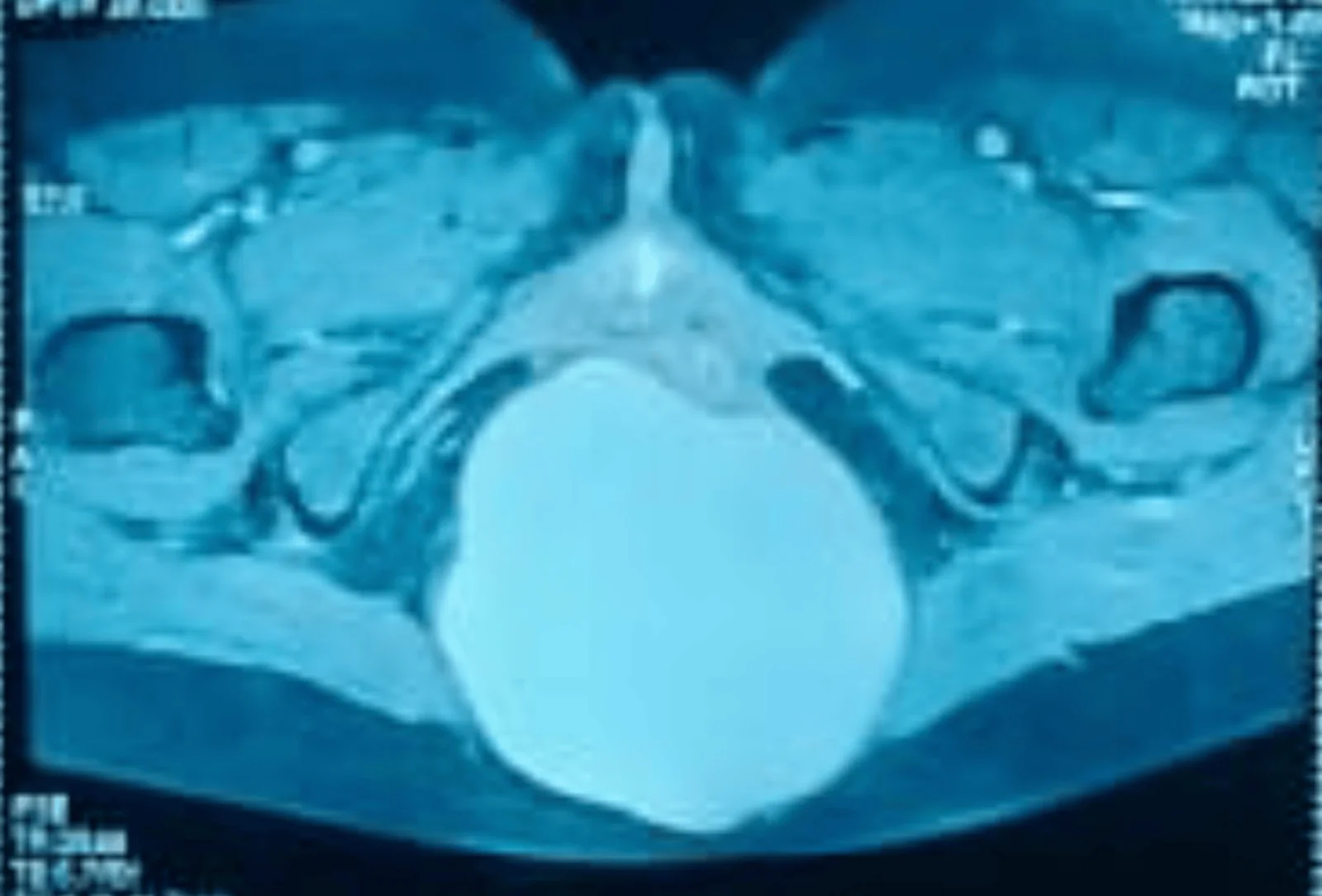
MRI revealing large retrococcygeal mass MRI: magnetic resonance imaging A well-circumscribed, markedly T2-hyperintense multiloculated lesion measuring 10 × 9 cm occupies the presacral/retrococcygeal space and displaces the rectum anteriorly. There is no communication with the rectal lumen or the spinal canal, and there is no sacral bony destruction, findings that argue against rectal duplication cyst, anterior sacral meningocele, and chordoma

Histopathology revealed a benign tailgut cyst lined by a combination of columnar, squamous, and transitional epithelium, and irregular smooth muscle bundles in the cyst wall, confirming the diagnosis. No evidence of malignancy was observed (Figure 3) [4,8].

**Figure 3:**
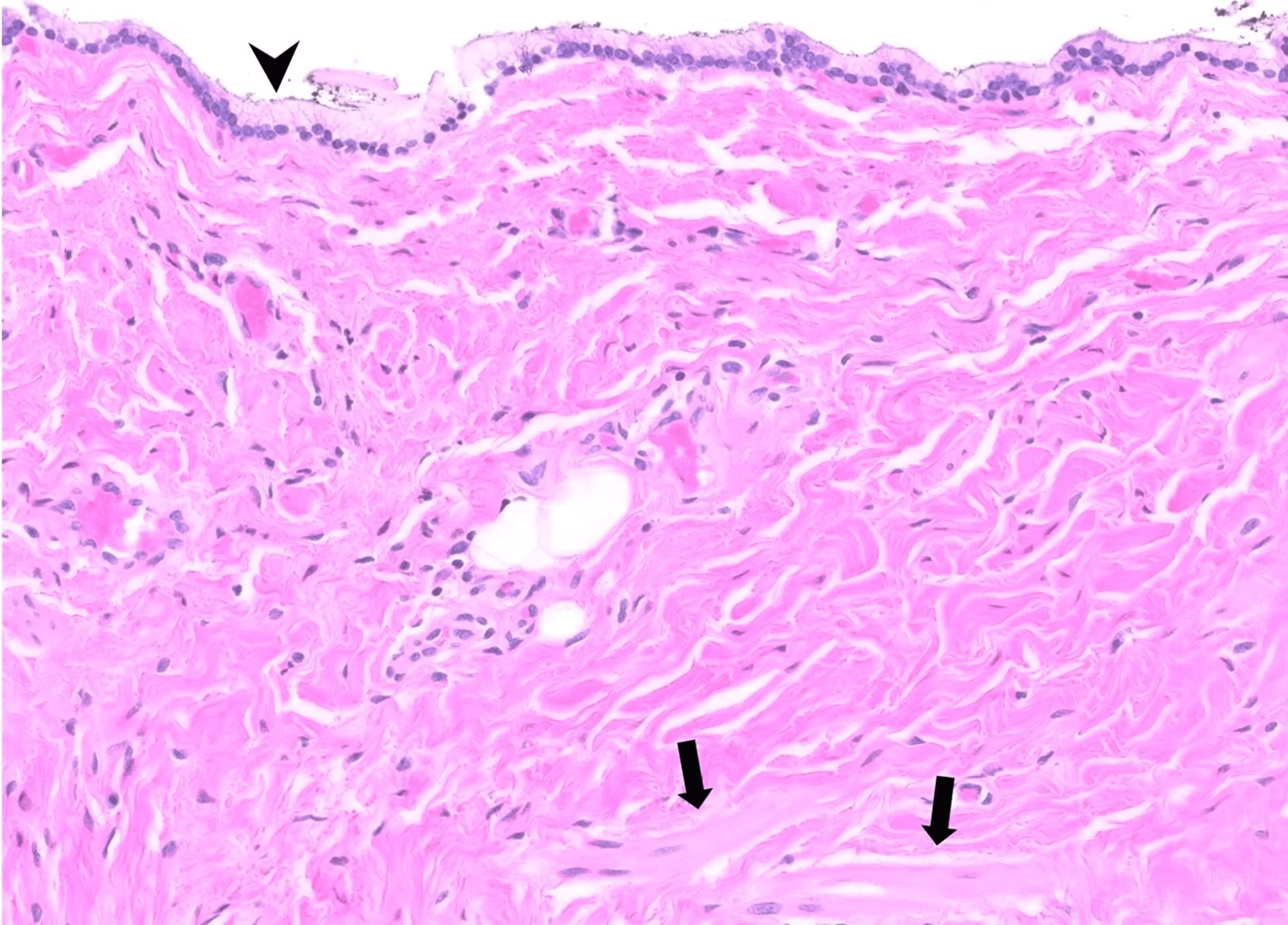
Histopathology of benign tailgut cyst High-power magnification shows a tailgut cyst lined by mucinous columnar epithelium (arrowhead) and irregularly arranged bundles of smooth muscle in the cyst wall (arrows). There is no cytological atypia, mitotic activity, or evidence of malignancy

Surgical management

The patient underwent elective surgical excision under general anesthesia. Intraoperatively, a 10 × 9 cm retrococcygeal cyst was identified and excised en bloc with meticulous dissection to preserve adjacent structures (Figure 4). The cyst was delivered intact, without spillage of its contents. No communication with the rectum or spinal canal was observed.

**Figure 4:**
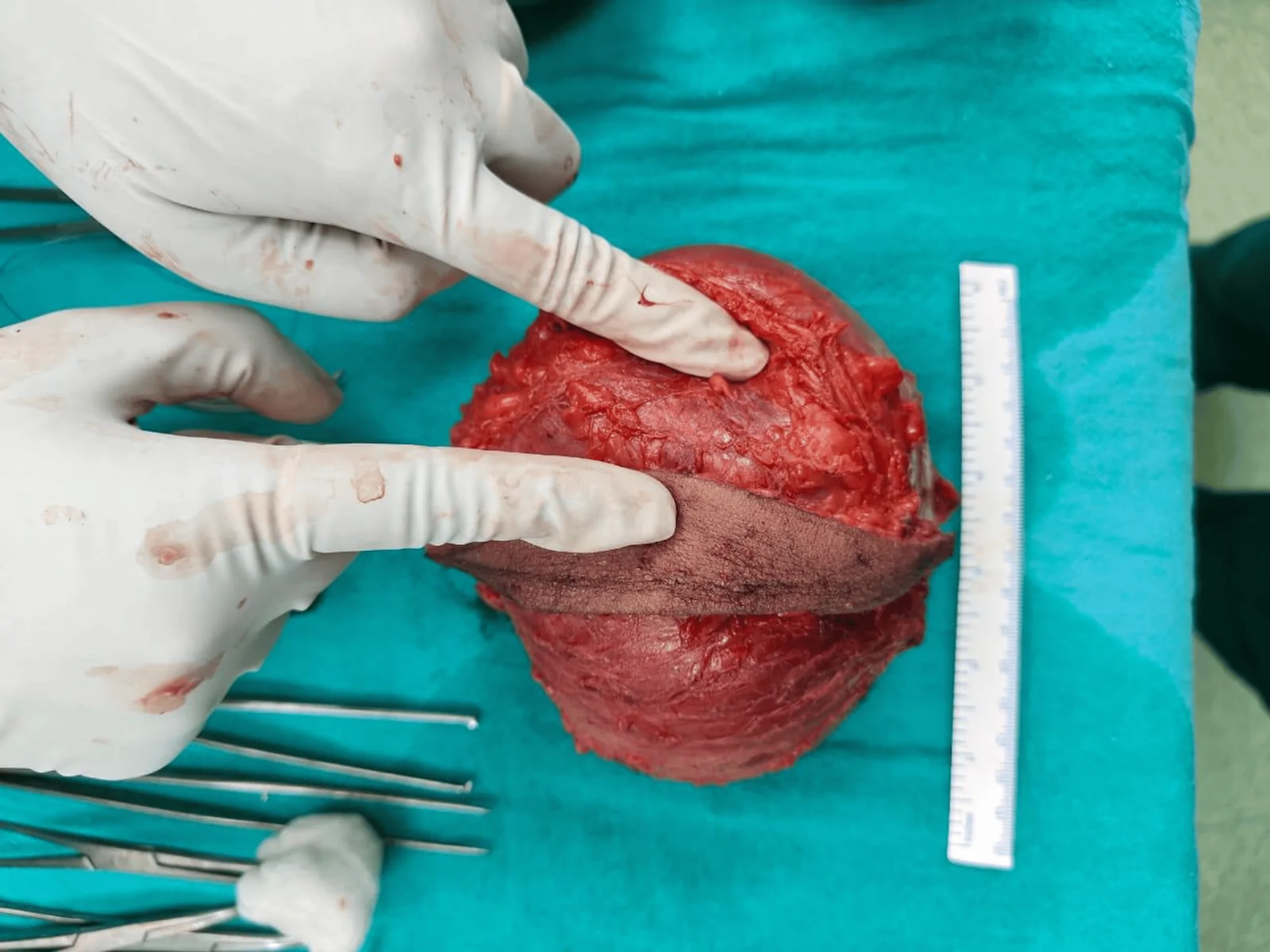
Retrococcygeal cyst The cyst is seen after en bloc dissection through the posterior retrococcygeal approach, delivered intact with an unruptured wall and preserved capsule. A scale placed alongside demonstrates the maximum dimensions of 10 × 9 cm

Postoperative course

The patient was managed with intravenous antibiotics, analgesics, antacids, and bowel regulation. The postoperative course was uneventful, with normal wound healing and bowel function. The patient was discharged on postoperative day 7 with instructions for regular dressing changes, a high-fiber diet, and outpatient follow-up. She was reviewed in the outpatient clinic at two weeks, one month, three months, six months, one year, and 18 months after surgery. The wound healed primarily, and she reported complete resolution of the gluteal swelling and discomfort, with normal bowel and urinary function and no neurological deficit. At the most recent review, 18 months after operation, she remained asymptomatic with no clinical evidence of recurrence. 

## Discussion

Tailgut cysts are rare, with an estimated incidence of one in 40,000 hospital admissions [9]. They predominantly occur in the retrorectal space and are four times more common in females [10]. In the largest reported series, most patients presented in the fourth and fifth decades of life, and our 35-year-old patient falls within this range [1]. Because of their deep pelvic location and nonspecific symptoms, the diagnosis is often delayed or incidental [11]. Our patient had been aware of the swelling for five years before definitive treatment, and this long lag is the likely reason the lesion reached 10 × 9 cm, considerably larger than the majority of reported tailgut cysts, which usually measure only a few centimeters [1].

The differential diagnosis of a retrorectal or gluteal cystic mass is wide and includes epidermoid and dermoid cysts, rectal duplication cyst, anterior sacral meningocele, sacrococcygeal teratoma, chordoma, neurogenic tumors, and chronic infected pilonidal disease [12]. In our patient, the absence of a midline sinus or discharge argued against pilonidal disease, the smooth fluctuant character and long indolent history argued against a neoplastic solid lesion, and an unremarkable digital rectal examination with no neurological deficit made a lesion arising from the rectal wall or sacral canal unlikely. These clinical impressions were confirmed on imaging.

MRI is the imaging modality of choice, offering detailed visualization of lesion extent and relation to adjacent structures [12]. Reported diagnostic features include high T2-weighted signal intensity, internal septations, and a definitive presacral location [8]. In our case, MRI findings of a large retrorectal cyst displacing the rectum without bowel or spinal communication strongly favored a tailgut cyst; the absence of communication with the rectal lumen excluded a duplication cyst, the absence of a dural connection and of the characteristic scimitar sacrum excluded an anterior sacral meningocele, and the absence of bony destruction or a solid enhancing component excluded chordoma [6]. Preoperative percutaneous biopsy was not performed and is generally discouraged in presacral lesions because of the risk of infection, fistula formation, and seeding along the biopsy track [7].

Complete surgical excision remains the standard of care to prevent complications such as infection, recurrence, or rare malignant transformation [13]. The operative approach is dictated largely by the level of the lesion: a posterior (retrococcygeal or Kraske) approach is appropriate for lesions lying below the level of S3, an anterior abdominal approach for those extending above it, and a combined abdominoperineal approach for very large or extensive lesions [7]. The retrococcygeal approach remains the gold standard for low lesions, with 92% success rates in complete excision [7]. Minimally invasive techniques may be considered for smaller lesions [14]. In our patient, the cyst was low-lying and predominantly retrococcygeal, and a posterior approach was therefore chosen; it gave direct access to the lower pole of the cyst, allowed the plane between the cyst and the rectum to be developed under direct vision, and avoided a laparotomy. The cyst was delivered intact, which matters because rupture and incomplete excision are the principal determinants of local recurrence [13].

Histopathology confirms the diagnosis, typically revealing a multiloculated cyst lined by various epithelial types, reflecting its embryologic origin [4]. Our case demonstrated columnar, squamous, and transitional epithelium without malignant features, together with irregular, haphazardly arranged bundles of smooth muscle within the cyst wall. This disorganized muscle is itself a useful diagnostic point, since a rectal duplication cyst, the closest histological mimic, has a well-formed, layered muscularis propria and is lined by a single type of intestinal epithelium [10].

The most important reason for complete excision is the risk of malignant change, reported in 3-5% of cases, with adenocarcinoma the commonest histological type [9]. Malignancy is more likely in long-standing and larger lesions, and features that should raise suspicion include recent rapid growth, new pain, and solid or enhancing components on MRI [3]. Careful sampling of the entire cyst wall at histopathological examination is therefore essential, as neoplastic change may be focal and confined to a small area of the lining [8]. Our specimen was reported free of atypia throughout, and the five-year history of a stable, painless swelling was reassuring in retrospect. Malignant risk has been the principal argument advanced in the literature for excising even asymptomatic tailgut cysts [5].

Reported outcomes after complete excision are good, but recurrence after incomplete removal is well-documented, and periodic clinical follow-up is advised, particularly after large or ruptured lesions [13]. Cases in which a tailgut cyst was initially mistaken for an occult perianal mass emphasize how easily the diagnosis is missed when imaging is not obtained [14]. Our patient has remained free of recurrence at 18 months, which is reassuring, although late recurrence beyond this interval has been described and longer surveillance would be needed to exclude it entirely. The main limitation of this report is that it describes a single patient, so no general inference about outcome can be drawn; nonetheless, the case illustrates a complete diagnostic and therapeutic pathway from clinical suspicion through MRI to en bloc posterior excision and histological confirmation.

## Conclusions

Tailgut cysts, though rare, warrant prompt surgical management to prevent complications. This case highlights the importance of including tailgut cysts in the differential diagnosis of presacral or gluteal masses, with MRI serving as the imaging modality of choice for characterizing the lesion and planning the operative approach. Complete en bloc surgical excision, ideally without cyst rupture, remains the definitive treatment, ensuring favorable outcomes and minimizing recurrence risk.
